# Second-Line Medications for Women Aged 10 to 50 Years With Idiopathic Generalized Epilepsy

**DOI:** 10.1001/jamanetworkopen.2025.0354

**Published:** 2025-03-10

**Authors:** Emanuele Cerulli Irelli, Enrico Cocchi, Joanna Gesche, Javier Peña-Ceballos, Roberto H. Caraballo, Simona Lattanzi, Gionata Strigaro, Alessandra Morano, Patrick B. Moloney, Edoardo Ferlazzo, Angelo Pascarella, Adolfo Mazzeo, Alfredo D’Aniello, Chiara Pizzanelli, Chiara Milano, Loretta Giuliano, Veronica Viola, Barbara Mostacci, Francesco Fortunato, Patrizia Pulitano, Margherita Burani, Stefano Meletti, Pietro Pignatta, Marco Perulli, Domenica Battaglia, Eleonora Rosati, Norman Delanty, Giancarlo Di Gennaro, Antonio Gambardella, Angelo Labate, Francesca F. Operto, Anna T. Giallonardo, Christoph P. Beier, Carlo Di Bonaventura

**Affiliations:** 1 Department of Human Neurosciences, Sapienza University, Rome, Italy; 2 Department of Medical and Surgical Sciences, University of Bologna, 40126 Bologna, Italy; 3 Department of Neurology, Odense University Hospital, Odense, Denmark; 4 Department of Neurology, Beaumont Hospital, Dublin, Ireland; 5 Department of Neurology, Hospital de Pediatría Professor Dr Juan P. Garrahan, Buenos Aires, Argentina; 6 Neurological Clinic, Department of Experimental and Clinical Medicine, Marche Polytechnic University, Ancona, Italy; 7 Neurology Unit, Department of Translational Medicine, University of Piemonte Orientale, and Azienda Ospedaliero-Universitaria Maggiore della Carità, Novara, Italy; 8 Regional Epilepsy Centre, Bianchi-Melacrino-Morelli Great Metropolitan Hospital, Reggio Calabria, Italy; 9 Department of Medical and Surgical Sciences, Magna Graecia University of Catanzaro, Catanzaro, Italy; 10 IRCCS NEUROMED, Pozzilli, Isernia, Italy; 11 Neurology Unit, Department of Clinical and Experimental Medicine, University of Pisa, Pisa, Italy; 12 Department of Medical and Surgical Sciences and Advanced Technologies G.F. Ingrassia, Section of Neurosciences, University of Catania, Catania, Italy; 13 IRCCS Istituto delle Scienze Neurologiche di Bologna, Bologna, Italy- Full member of the EERN EpiCARE; 14 Institute of Neurology, University Magna Graecia, Catanzaro, Italy; 15 Department of Biomedical, Metabolic and Neural Science, Center for Neuroscience and Neurotechnology, University of Modena and Reggio Emilia, Modena, Italy; 16 Humanitas Gradenigo Hospital, Turin, Italy; 17 Fondazione Policlinico Universitario Agostino Gemelli IRCCS, Rome, Italy; 18 Neuromuscular and Sense Organs Department, Neurology 2, Careggi University Hospital, Florence, Italy; 19 School of Pharmacy and Biomolecular Sciences, Royal College of Surgeons in Ireland, Dublin, Ireland; 20 FutureNeuro, the Science Foundation Ireland Research Centre for Chronic and Rare Neurological Diseases, Dublin, Ireland; 21 Neurophysiopatology and Movement Disorders Clinic, University of Messina, Italy; 22 Department of Science of Health, School of Medicine, University of Catanzaro, Catanzaro, Italy; 23 AOU Policlinico Umberto I, Rome, Italy; 24 Department of Clinical Research, University of Southern Denmark, Odense, Denmark

## Abstract

**Importance:**

Women with idiopathic generalized epilepsy (IGE) face challenges in treatment due to limited options that are both effective and safe.

**Objective:**

To evaluate the effectiveness and safety of substitution monotherapy vs add-on therapy as second-line options for women who might become pregnant with IGE after failure of first-line antiseizure medications (ASMs) other than valproic acid.

**Design, Setting, and Participants:**

Multicenter retrospective comparative effectiveness cohort study at 18 primary, secondary, and tertiary adult and children epilepsy centers across 4 countries, analyzing data from 1995 to 2023. Participants were women aged 10 to 50 years diagnosed with IGE who were prescribed a second line of ASM.

**Main Outcomes and Measures:**

Treatment failure (TF), defined as the replacement or addition of a second ASM due to ineffectiveness, was compared between patients receiving ASM add-on or substitution monotherapy using inverse probability of treatment weighting (IPTW)–adjusted Cox proportional hazards regression. Exploratory analyses were also conducted to assess the effectiveness of individual ASMs and various ASM combinations.

**Results:**

This study included 249 women with a median (IQR) age of 18.0 (15.5-22.0) years. Among them, 146 (58.6%) received an add-on regimen, and 103 (41.4%) received substitution monotherapy. During follow-up, TF occurred in 48 patients (32.9%) receiving add-on therapy and 36 (35.0%) using substitution monotherapy, with no significant differences between groups (IPTW-adjusted hazard ratio [HR], 0.89; 95% CI, 0.53-1.51; *P* = .69). ASM discontinuation due to ineffectiveness or adverse effects occurred in 36 patients (24.7%) receiving add-on therapy and 29 (28.2%) receiving substitution monotherapy, showing no significant differences (IPTW-adjusted HR, 0.97; 95% CI, 0.57-1.65; *P* = .92). Rates of ASM discontinuation due to adverse effects only were low in both groups, occurring in 13 patients (9.0%) receiving add-on therapy and 9 (8.7%) receiving a substitution monotherapy. Among add-on regimens other than valproic acid, the combination of levetiracetam and lamotrigine demonstrated a lower risk of TF compared with other combinations with levetiracetam plus other ASM (adjusted HR, 2.41; 95% CI, 1.12-5.17; *P* = .02) and lamotrigine plus other ASM (adjusted HR, 4.03; 95% CI, 1.73-9.39; *P* = .001). However, valproic acid remained the most effective second-line ASM when considering individual agents.

**Conclusions and relevance:**

In this comparative effectiveness study of second-line treatment strategies for women with IGE, no significant differences were observed between substitution monotherapy and add-on therapy.

## Key Points

**Question:**

What are the effectiveness and safety of substitution monotherapy vs add-on therapy as second-line treatment options for women with idiopathic generalized epilepsy (IGE) after failure of first-line antiseizure medications (ASMs) other than valproic acid?

**Findings:**

This comparative effectiveness study of 249 women with IGE found no significant differences in effectiveness or safety between substitution monotherapy and add-on therapy. Among non–valproic acid add-on regimens, the levetiracetam and lamotrigine combination demonstrated superior effectiveness compared with other ASM combinations.

**Meaning:**

These findings suggest both substitution monotherapy and add-on therapy are viable second-line treatment strategies for women with IGE.

## Teaser

This comparative effectiveness study evaluates effectiveness and safety of substitution monotherapy vs add-on therapy as second-line options for women who might become pregnant with idiopathic generalized epilepsy after failure of first-line antiseizure medications other than valproic acid.

## Introduction

Idiopathic generalized epilepsy (IGE) is a frequent form of epilepsy, accounting for approximately 15% to 20% of all cases seen in epilepsy outpatient clinics, and it exhibits a slight female predominance.^1^ Valproic acid has traditionally been the preferred treatment for IGE due to its broad efficacy across various generalized seizure types.^2,3^ However, the use of valproic acid in women who might become pregnant is strongly discouraged due to the significant risks of major congenital malformations and neurodevelopmental disorders in exposed offspring.^4,5^ This poses a significant challenge in finding effective and safe alternative antiseizure medications (ASMs) for this population.^6,7^

In previous research, this study group compared the effectiveness of levetiracetam and lamotrigine—2 ASMs with the lowest teratogenic potential—as initial monotherapies in women with IGE. The results highlighted levetiracetam as the preferred initial monotherapy for juvenile myoclonic epilepsy (JME) and lamotrigine for juvenile absence epilepsy (JAE), while both ASMs demonstrated similar effectiveness for epilepsy with generalized tonic-clonic seizures alone (GTCA).^8,9^

However, more than half of patients with epilepsy do not achieve adequate seizure control with first-line ASM monotherapy, necessitating a second ASM regimen.^10^ Current evidence guiding the choice between substitution monotherapy and add-on therapy (combination therapy) after the failure of initial monotherapy is limited and inconclusive.^11^ Some observational studies report conflicting outcomes, while a few small randomized clinical trials (RCTs) indicate similar seizure-free rates between substitution and combination therapies.^12,13,14,15^ This uncertainty is further complicated for women who might become pregnant, who must face the additional constraint of avoiding valproic acid. Given this background, there is a critical need for evidence-based treatment strategies tailored to this specific population to guide clinicians through the complex therapeutic landscape for women with IGE.

This study examines the effectiveness of different management strategies for women with IGE who did not achieve seizure control with a first-line ASM other than valproic acid. Specifically, we compare the efficacy and safety of substitution monotherapy vs add-on therapy after the failure of first-line ASMs other than valproic acid. Additionally, we explore the comparative effectiveness of specific ASMs and different ASM combinations following the failure of the first ASM.

## Methods

### Study Participants

This multicenter retrospective comparative effectiveness study was conducted and reported according to the Strengthening the Reporting of Observational Studies in Epidemiology (STROBE) and International Society for Pharmacoeconomics and Outcomes Research (ISPOR) reporting guidelines.^16,17^ Local ethics committees at each institution approved the study and written informed consent was obtained by all participants.

The study cohort was identified from a population of patients attending 18 primary, secondary, and tertiary adult and children epilepsy centers across 4 countries from 1995 to 2023. Eligibility criteria were: (1) female sex; (2) diagnosis of IGE per International League Against Epilepsy criteria^18^; (3) prescription of an ASM effective on generalized seizures alternative to valproic acid as initial monotherapy during childbearing age (10 to 50 years), including levetiracetam, lamotrigine, topiramate, zonisamide, or ethosuximide; (4) failure of initial monotherapy due to ineffectiveness; (5) prescription of a second ASM, either as add-on or substitution monotherapy; and (6) a minimum follow-up of 12 months after second ASM prescription, unless primary outcome occurred earlier.

Patient demographics, baseline clinical characteristics, and treatment details regarding the first-line ASM regimen were extracted from clinical records and electronic health records following the methods outlined in our previous works.^8,9^ Data on the second ASM regimen, including type, dosage, and whether it was used as an add-on or as substitution monotherapy, were collected, along with adverse effects leading to second ASM discontinuation.

### Outcome Measures

The primary outcome was the time from the initiation of a second-line ASM to treatment failure (TF) of this second ASM due to ineffectiveness. TF was defined as the need to switch to another ASM or to add an adjunctive ASM because of a lack of adequate response. If the second ASM was discontinued due to considerations for future pregnancy or for adverse effects, the event was not counted as TF due to ineffectiveness, and the patient was censored from the analysis of the primary outcome. Secondary outcomes included treatment retention, measured as the time from the prescription of the second ASM until its discontinuation due to ineffectiveness or adverse effects, and seizure freedom, considering patients who achieved this outcome during treatment with their second ASM (ie, without experiencing a previous TF).^19^

### Statistical Analysis

The complete statistical analysis plan has been fully detailed in eMethods in Supplement 1. Multiple imputation with chained equations was used to impute missing data, as explained in previous studies.^20^ The pattern and rate of missing data are represented in eFigure 1 in Supplement 1.

Consistent with our previous studies,^8,9^ we applied inverse probability of treatment weighting (IPTW) to adjust for potential selection biases associated with second ASM assignment. The IPTW was calculated as the reciprocal of the probability of receiving either an add-on regimen or a substitution monotherapy using a multivariable logistic regression model. Based on previous literature,^21,22,23^ this model incorporated multiple covariates including age at second ASM prescription, febrile seizures, epilepsy syndrome, catamenial worsening of seizures, history of status epilepticus, mild intellectual disability, and use of valproic acid as second ASM regimen. Age was treated nonlinearly using a restricted cubic spline with knots at the 1st, 25th, 50th, 75th, and 99th percentiles based on findings from previous studies and to mitigate potential effects of linearity assumption violations.^7,8,9^

An IPTW-adjusted Cox proportional hazards model was employed to assess the differences in time to TF between substitution monotherapy and add-on as second ASM regimen. The time of entry was the date of second ASM prescription, and the time of end point was the date of TF or the last follow-up visit, truncated at 5 years of follow-up. To address potential differences associated with the year of prescription over the extended study timespan, a sensitivity analysis was conducted using a mixed IPTW-adjusted Cox proportional hazards model, with the year of prescription incorporated as a random effect. Additionally, a further sensitivity analysis of the primary outcome was performed after excluding patients using valproic acid as second ASM.

Regarding secondary outcome measures, the same IPTW-weighted Cox proportional hazards model was used to analyze the differences in ASM retention. For estimating differences in seizure freedom between the 2 ASM regimens, an IPTW-weighted binary logistic regression analysis was conducted, with follow-up duration included as an additional covariate to account for variations in follow-up.

Additionally, we compared (1) TF due to ineffectiveness based on specific ASMs prescribed after the failure of the first-line monotherapy assessed through multivariable Cox regression, using the type of ASM regimen (ie, substitution monotherapy or add-on) and epilepsy syndrome as covariate. To ensure more reliable estimates, only ASMs prescribed to at least 10 patients were included in this analysis; (2) TF based on specific ASM combinations among patients using add-on regimens assessed through multivariable Cox regression analysis and including epilepsy syndrome as covariate; (3) TF according to specific IGE syndromes using multivariable Cox regression adjusted for the prescribed ASM.

Finally, the proportion of patients experiencing ASM discontinuation due to adverse effects in the 2 treatment groups was compared through Fisher exact test. Two-sided *P* values less than .05 were considered statistically significant. All analyses in this study were conducted using R version 4.3.3 (R Project for Statistical Computing).

## Results

### Baseline Clinical Characteristics

Among 664 female patients with IGE aged 10 to 50 years who were prescribed an ASM alternative to valproic acid as first-line monotherapy, 265 (39.9%) received a second ASM after TF due to ineffectiveness. Sixteen (6.0%) of these patients were excluded due to insufficient follow-up after the second ASM prescription. The final study population thus included 249 patients with a median (IQR) age of 18.0 (15.5-22.0) years at the time of the second ASM prescription and a median (IQR) follow-up duration of 60 (24-60) months. A study flowchart illustrating patient selection and the study design is provided in eFigure 2 in Supplement 1. Regarding observed IGE syndromes, JME was the most frequently diagnosed, with 105 patients (42.2%), followed by absence epilepsy in 79 patients (31.7%; 1 diagnosed with childhood absence epilepsy and 78 with JAE), and GTCA in 65 (26.1%). Regarding ASMs used as first-line monotherapies, 109 of 249 patients (43.8%) were prescribed levetiracetam, 108 (43.4%) lamotrigine, 18 (7.2%) topiramate, 10 (4%) ethosuximide, and 4 (1.6%) zonisamide.

### Second-Line Treatment Patterns

After failure of the first-line monotherapy, 146 women (58.6%) were prescribed an add-on regimen, and 103 (41.4%) were prescribed substitution monotherapy (see eFigure 3 in Supplement 1 for a representation of the year of second ASM prescription, along with the type of treatment regimen used during different periods). A comparison of baseline characteristics in both treatment groups before and after IPTW balancing is described in the Table, with standardized mean differences before and after balancing fully reported in eFigure 4 in Supplement 1.

**Table.** Patient Clinical Characteristics According to Treatment Regimen

Variable	Patients, No. (%)		*P* value	IPTW-adjusted *P* value
	Add-on	Substitution		
Patients, No.	146	103	NA	NA
Age at second ASM prescription, mean (SD), y	20.13 (6.6)	18.59 (5.5)	.06	.68
History of febrile seizures	16 (11.0)	14 (13.6)	.67	.68
Mild intellectual disability	10 (6.8)	8 (7.8)	.98	.67
Epilepsy syndrome				
Absence epilepsy	39 (33.6)	30 (29.1)	.49	.93
Juvenile myoclonic epilepsy	57 (39.0)	48 (46.6)		
Epilepsy with GTCS alone	40 (27.4)	25 (24.3)		
Previous status epilepticus	8 (8.6)	6 (4.3)	.29	.65
Catamenial worsening of seizures	33 (22.9)	19 (18.4)	.49	.98
Use of valproic acid as second ASM	22 (21.4)	26 (17.8)	.59	.70

Abbreviations: ASM, antiseizure medication; GTCS, generalized tonic-clonic seizures; IPTW, inverse probability treatment weighting; NA, not applicable.

**Figure 1.**
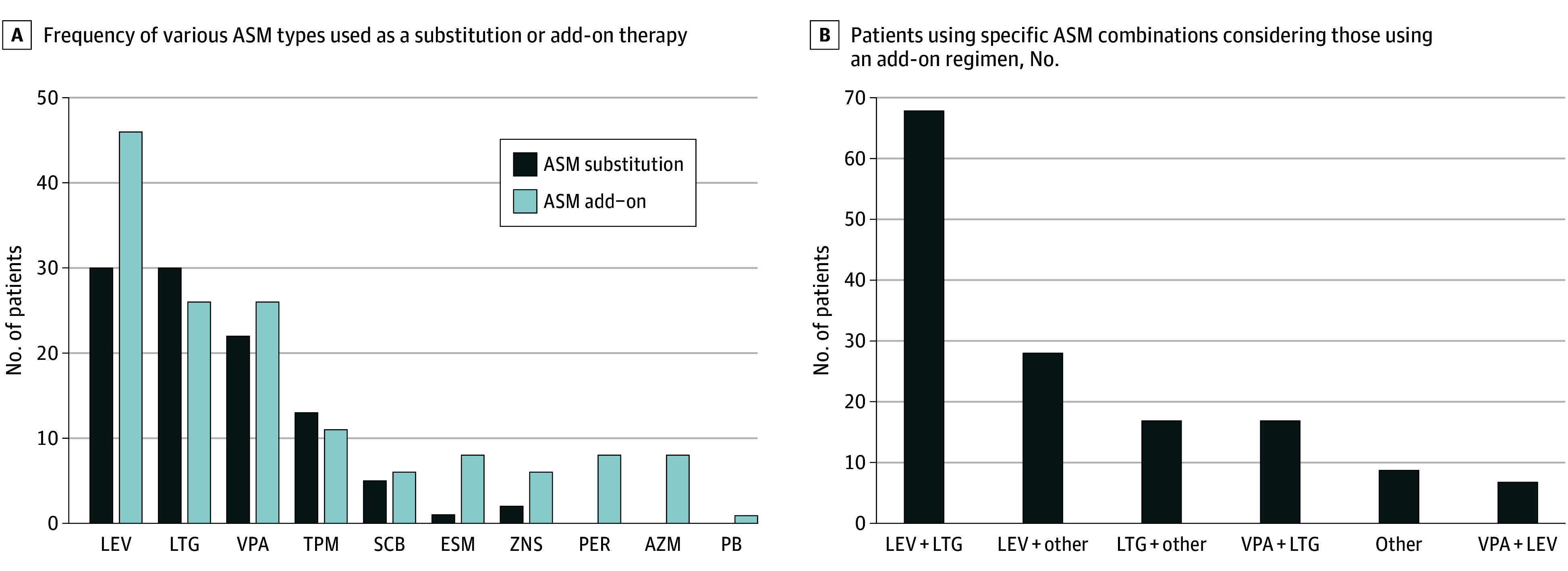
Overview of Second Antiseizure Medication (ASM) Types and Combination Therapies AZM indicates acetazolamide; ESM, ethosuximide; LEV, levetiracetam; LTG, lamotrigine; PB, phenobarbital; PER, perampanel; SCB, sodium channel blocker (includes 8 lacosamide and 3 carbamazepine); TPM, topiramate; VPA, valproic acid; ZNS, zonisamide.

When considering the second ASM regimen, the most commonly used ASM was levetiracetam in 76 women (30.5%), followed by lamotrigine in 56 (22.5%), valproic acid in 48 (19.3%), and topiramate in 24 (9.6%). The full range of ASMs used as second-line treatments, along with their prescribed regimens (ie, as add-on therapy or substitution monotherapy), are represented in Figure 1. Among those receiving an add-on regimen, the most frequent ASM combination was levetiracetam plus lamotrigine, used by 68 of 146 women (46.6%). All prescribed ASM combinations are represented in Figure 1.

### Comparative Effectiveness of Add-On Therapy and Substitution Monotherapy

**Figure 2.**
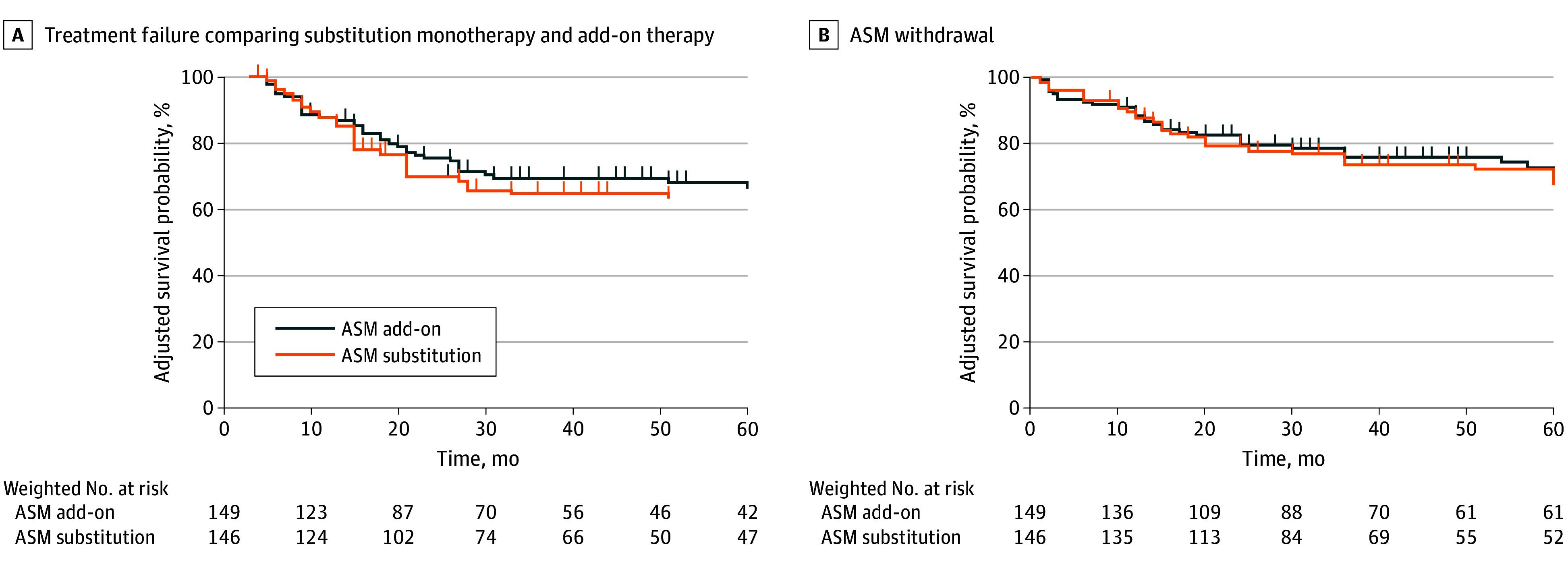
Inverse Probability of Treatment Weighting (IPTW)–Adjusted Curves of Treatment Failure and Antiseizure Medication (ASM) Withdrawal Between Add-On Regimen and Substitution Monotherapy A, IPTW-adjusted survival curves; B, IPTW-adjusted survival curves, considering both adverse effects and ineffectiveness.

After the failure of a first ASM alternative to valproic acid, there were 84 TF events (33.7%) overall during follow-up, and this proportion increased to 76 out of 201 patients (37.8%) when considering those who were not prescribed valproic acid as a second ASM. In the primary outcome analysis, TF occurred in 48 of 146 participants receiving an add-on regimen (32.9%) and in 36 of 103 using substitution monotherapy (35%), respectively (unadjusted hazard ratio [HR], 0.92; 95% CI, 0.58-1.46; *P* = .92) (Figure 2A). The IPTW-weighted Cox model confirmed the lack of significant differences between the 2 ASM regimens after adjustment for all baseline variables (IPTW-adjusted HR, 0.89; 95% CI, 0.53-1.51; *P* = .69) (Figure 2). In line, sensitivity analysis of the primary outcome including year of prescription as random effect yielded similar results (IPTW-adjusted HR, 0.94; 95% CI, 0.62-1.43; *P* = .78). Additional sensitivity analysis excluding patients using valproic acid as second ASM further confirmed the lack of statistically significant differences between add-on and substitution monotherapy regimens (IPTW-adjusted HR, 1.35; 95% CI, 0.80-2.30; *P* = .26).

When considering secondary outcomes, ASM withdrawal due to ineffectiveness or adverse effects occurred in 36 patients (24.7%) using an add-on regimen and 29 patients (28.2%) prescribed a substitution monotherapy, with no significant differences between the 2 groups (IPTW-adjusted HR, 0.97; 95% CI, 0.57-1.65; *P* = .92) (Figure 2B). Seizure freedom with the second ASM occurred in 68 patients (50.4%) using an add-on regimen and in 60 (58.8%) receiving a substitution monotherapy, showing no significant differences (IPTW-adjusted odds ratio, 0.80; 95% CI, 0.44-1.45; *P* = .47). The results for primary and secondary outcomes were consistent when recalculating the IPTW under the assumption of a linear association between age and treatment regimen (eTables 1, 2, and 3 in Supplement 1).

In the exploratory analysis of specific IGE syndromes, the add-on regimen had a nonsignificant lower rate of TF in patients with GTCA (adjusted HR, 0.27; 95% CI, 0.06-1.19; *P* = .08) and JME (adjusted HR, 0.81; 95% CI, 0.41-1.63; *P* = .56) compared with substitution monotherapy, whereas nominally higher rates were observed in those with JAE (adjusted HR, 1.53; 95% CI, 0.56-4.18; *P* = .40). See eTables 4, 5, and 6 in Supplement 1 for detailed results of the multivariable Cox regression analysis models.

### Comparative Effectiveness of Individual ASMs and Specific ASM Combinations

**Figure 3.**
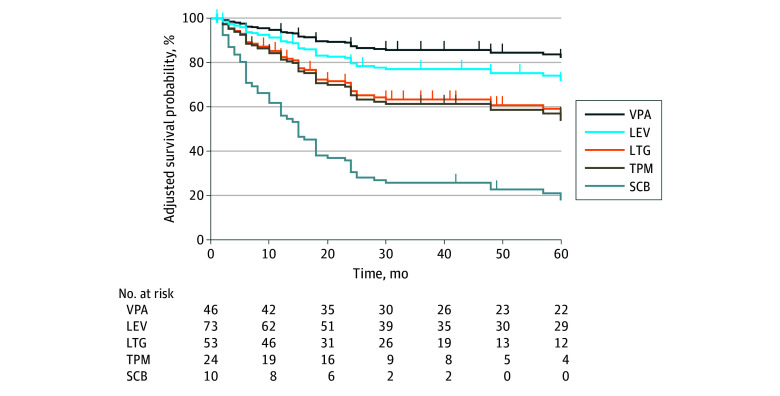
Adjusted Time-to-Event Curves of Treatment Failure Based on Specific Antiseizure Medications Used as Second Regimen Survival curves for treatment failure based on specific antiseizure medications (ASMs) used as the second regimen, either as an add-on or substitution monotherapy, adjusted for epilepsy syndrome and the type of regimen used. Only ASMs prescribed to at least 10 patients are included. Each ASM is represented by a specific color, compared with reference ASM (ie, valproic acid). LEV indicates levetiracetam; LTG, lamotrigine; SCB, sodium channel blocker (8 lacosamide, 3 carbamazepine); TPM, topiramate; VPA, valproic acid.

When evaluating individual agents, whether prescribed as alternative monotherapy or as part of an add-on regimen, valproic acid was associated with a significantly lower risk of TF (8 of 48 patients [16.7%]) compared with all other ASMs (76 of 201 patients [37.8%]), except for levetiracetam (adjusted HR, 1.75; 95% CI, 0.69–4.50; *P* = .24) (Figure 3). Detailed results of the multivariable Cox regression analysis are reported in eTable 7 in Supplement 1. A full comparison, also including ASMs prescribed to fewer than 10 patients, has been illustrated in eFigure 5 in Supplement 1.

**Figure 4.**
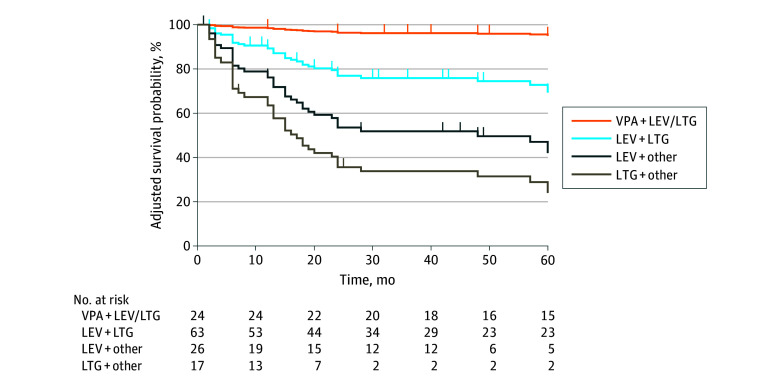
Adjusted Time-to-Event Curves of Treatment Failure Based on Specific Combinations of Antiseizure Medications in Patients Using an Add-On Regimen Survival curves for treatment failure based on specific antiseizure medication (ASM) combination, adjusted for epilepsy syndrome. Each ASM combination is represented by a specific color, compared with reference ASM combination (ie, lamotrigine [LTG] plus levetiracetam [LEV]). VPA indicates valproic acid.

When considering ASM combinations among patients using an add-on regimen, levetiracetam plus lamotrigine was associated with a significantly reduced risk of TF compared with levetiracetam plus other (ie, any other ASM) (adjusted HR, 2.41; 95% CI, 1.12-5.17; *P* = .02) and lamotrigine plus other (adjusted HR, 4.03; 95% CI, 1.73-9.39; *P* = .001), whereas levetiracetam plus lamotrigine showed nonsignificantly lower effectiveness compared with valproic acid plus levetiracetam or valproic acid plus lamotrigine (adjusted HR, 0.14; 95% CI, 0.02-1.07; *P* = .06) (Figure 4). Detailed results of the multivariable Cox regression analysis are reported in eTable 8 in Supplement 1.

### Adverse Effects

ASM withdrawal due to adverse effects only occurred in 22 patients (8.9%) overall. Specifically, 13 patients (9.0%) using an add-on regimen discontinued the prescribed ASM due to adverse effects and 9 (8.7%) using a substitution monotherapy, with no significant differences between groups. Additionally, no significant differences emerged among different types of ASM, with higher rates of discontinuation due to adverse effects occurring for topiramate (3 of 24 patients [12.5%]), levetiracetam (9 of 76 patients [11.8%]), and valproic acid (5 of 47 patients [10.6%]).

## Discussion

This study provides critical insights into the management of IGE in women who might become pregnant, a group for whom clinical care poses significant challenges due to the limited number of ASMs that are both effective and safe. Indeed, after the failure of a first-line ASM alternative to valproic acid, there is limited knowledge about the most effective treatment strategies, including the choice of ASM regimen, potential combinations, and specific ASMs to use in this population.

Notably, this is the first study to directly compare the effectiveness of substitution monotherapy vs add-on therapy as a second ASM regimen in women with IGE, employing an IPTW approach to control for potential confounding factors. Previous studies have primarily focused on mixed cohorts of men and women with both focal and generalized epilepsy, reporting similar effectiveness and adverse effect profiles between alternative monotherapy and early add-on therapy.^12,13^ Similarly, 2 RCTs in focal epilepsy demonstrated comparable outcomes between these treatment strategies.^14,15^ However, no prior study has specifically addressed patients with IGE, who are generally considered to have a lower likelihood of drug resistance compared with those with focal epilepsy,^24^ leading to uncertainty whether a more proactive add-on regimen approach would improve the chances of treatment success in this group. Our findings showed that both substitution monotherapy and add-on therapy did not show significant differences in terms of TF, ASM retention, seizure freedom, and discontinuation rates due to adverse effects. The absence of a significant difference suggests that, in the context of a second-line regimen after the failure of the first ASM, either approach could be considered viable among women with IGE. This enables clinicians to make personalized treatment decisions based on individual patient profiles, preferences, and potential adverse effects.

Importantly, this study also provided a comparative analysis on the effectiveness of different ASM combinations among patients using add-on regimens. Among these patients, levetiracetam combined with lamotrigine emerged as the most commonly prescribed option, accounting for approximately half of women receiving a combination therapy. A recent study by Cohen and colleagues^25^ showed that the levetiracetam plus lamotrigine combination is associated with a low rate of major congenital malformations, which was significantly lower than valproic acid monotherapy. Additionally, a retrospective study evaluating the cognitive performance in patients treated with polytherapy regimens highlighted that the levetiracetam plus lamotrigine combination was associated with a particularly favorable cognitive profile.^26^ This study further supports the use of this combination therapy in women with IGE, highlighting its effectiveness profile. Specifically, this comparative effectiveness analysis demonstrated a lower risk of TF with levetiracetam plus lamotrigine compared with either levetiracetam or lamotrigine combined with other ASMs.

Additionally, this study also evaluated TF due to ineffectiveness based on specific ASMs used as second-line treatments, either as alternative monotherapies or add-on regimens. valproic acid was confirmed as the most effective ASM, even in this specific clinical scenario, reinforcing its superior effectiveness compared with other ASMs in patients with IGE as reported in previous studies.^27,28^ By significantly reducing the likelihood of failure due to ineffectiveness with a second ASM regimen (37.8% vs 16.7%), valproic acid appears to prevent the development of drug-resistant epilepsy, thereby potentially mitigating the well-known psychosocial and health burdens associated with this condition.^29^ In this regard, a recent population-based study highlighted that patients with epilepsy who withdraw from or substitute valproic acid may face a higher risk of injuries, hospitalizations, and other negative health outcomes, although no significant differences were found in terms of mortality.^30^ However, despite its effectiveness, valproic acid is burdened by substantial teratogenic effects, with even low doses doubling the likelihood of major congenital malformations compared with levetiracetam and lamotrigine.^4^ In this context, for female patients with IGE experiencing severe or disabling seizures—particularly those at risk of sudden unexpected death in epilepsy or serious seizure-related injuries—the decision to use valproic acid should involve a shared decision-making process, ensuring that women are fully informed and that their motivations and perspectives are carefully considered.

Finally, in the exploratory analysis of specific IGE syndromes, a nonsignificant lower likelihood of TF was observed with add-on regimens in patients with GTCA and JME, while a higher rate of TF appeared to occur in JAE. Due to the limited number of patients diagnosed with each syndrome in our cohort, no definitive conclusions can be drawn due to the underpowered nature of these analyses. However, these findings suggest that future RCTs comparing these 2 treatment strategies among patients with IGE should consider focusing on specific IGE syndromes to provide more robust conclusions.

### Strengths and Limitations

Despite the strengths of this study, including a large, multicenter cohort and the use of an IPTW approach to mitigate bias, some limitations must be acknowledged. First, the retrospective nature of the study and reliance on existing medical records introduce the possibility of residual confounding. Second, the interval from second ASM initiation to the decision regarding subsequent ASM substitution or add-on may be influenced by the subjective decisions of individual physicians at each recruiting center. Additionally, while the use of IPTW helped balance the treatment groups, unmeasured confounders may still influence the results. Furthermore, the low prescription rates of less commonly used ASMs may have underpowered the exploratory analysis of the comparative effectiveness of specific ASMs. The relatively small sample size in these exploratory analyses also limited the number of covariates included in the multivariable Cox regression analysis. Furthermore, ASM combinations were grouped as levetiracetam plus others or lamotrigine plus others to avoid very small subgroups, which precluded comparisons of more specific ASM combinations. This limitation also prevented us from comparing the impact of combining different mechanisms of action on the choice and effectiveness of second-line ASM combinations.^31^

## Conclusions

In conclusion, this study provides valuable evidence to the ongoing debate regarding the optimal management of IGE in women who might become pregnant, particularly after the failure of first-line ASM therapy. Our findings support the use of either substitution monotherapy or add-on therapy as effective second-line options, with the choice best guided by individual patient characteristics. The combination of levetiracetam and lamotrigine emerges as a promising alternative for add-on therapy, offering a balance between efficacy and safety. However, when considering individual agents, valproic acid was confirmed as the most effective ASM even in this specific scenario. These findings aid in developing more informed and evidence-based treatment decisions in this challenging clinical context, offering clinicians valuable information for counseling their patients. Future research should aim to confirm these findings in RCTs, which would provide higher-level evidence and potentially uncover subtler differences between treatment strategies.

## Notes

Supplement 1.
eMethods.
**eTable 1. **IPTW-Weighted Models for Treatment Failure Assuming a Linear Relationship Between Age and Substitution Monotherapy or Add-on **eTable 2. **IPTW-Weighted Models for ASM Retention Assuming a Linear Relationship Between Age and Substitution Monotherapy/Add-on **eTable 3.** IPTW-Weighted Models for Seizure Freedom Assuming a Linear Relationship Between age and Substitution Monotherapy or Add-on **eTable 4. **Multivariable Cox Regression of Treatment Failure in Patients With Juvenile Absence Epilepsy **eTable 5.** Multivariable Cox Regression of Treatment Failure in Patients With Juvenile Myoclonic Epilepsy **eTable 6. **Multivariable Cox Regression of Treatment Failure in Patients With Epilepsy With Generalized Tonic-Clonic Seizures Alone **eTable 7.** Multivariable Cox Regression of Treatment Failure Comparing Different ASMs **eTable 8. **Multivariable Cox Regression of Treatment Failure Comparing Different ASM Combinations **eFigure 1.** Missingness of Variables and Combinations of Missingness Using the Intersecting Sets Visualization Method **eFigure 2.** Flowchart of Patient Selection, Second-Line Treatment Strategies, and Research Questions **eFigure 3.** Prescribing Pattern of Substitution Monotherapy or Add-on Therapy Across Different Time Periods **eFigure 4. **Love-Plot Showing Standardized Mean Difference (SMD) Between Add-on Regimen and Substitution Monotherapy Before and After Inverse Probability Treatment Weighting for all Covariates **eFigure 5. **Adjusted Time-to-Event Curves of Treatment Failure Based on Specific Antiseizure Medications (ASM) Used as Second Regimen
eReferences

Supplement 2.
Nonauthor Collaborators

Supplement 3.
Data Sharing Statement
