# Supplementary material for: Second-Line Medications for Women Aged 10 to 50 Years With Idiopathic Generalized Epilepsy
Source: JAMA Netw Open. 2025 Mar 10;8(3):e250354. doi: 10.1001/jamanetworkopen.2025.0354 (PMC11894492; doi:10.1001/jamanetworkopen.2025.0354)
Supplement: Supplement 1. — eMethods. eTable 1. IPTW-Weighted Models for Treatment Failure Assuming a Linear Relationship Between Age and Substitution Monotherapy or Add-on eTable 2. IPTW-Weighted Models for ASM Retention Assuming a Linear Relationship Between Age and Substitution Monotherapy/Add-on eTable 3. IPTW-Weighted Models for Seizure Freedom Assuming a Linear Relationship Between age and Substitution Monotherapy or Add-on eTable 4. Multivariable Cox Regression of Treatment Failure in Patients With Juvenile Absence Epilepsy eTable 5. Multivariable Cox Regression of Treatment Failure in Patients With Juvenile Myoclonic Epilepsy eTable 6. Multivariable Cox Regression of Treatment Failure in Patients With Epilepsy With Generalized Tonic-Clonic Seizures Alone eTable 7. Multivariable Cox Regression of Treatment Failure Comparing Different ASMs eTable 8. Multivariable Cox Regression of Treatment Failure Comparing Different ASM Combinations eFigure 1. Missingness of Variables and Combinations of Missingness Using the Intersecting Sets Visualization Method eFigure 2. Flowchart of Patient Selection, Second-Line Treatment Strategies, and Research Questions eFigure 3. Prescribing Pattern of Substitution Monotherapy or Add-on Therapy Across Different Time Periods eFigure 4. Love-Plot Showing Standardized Mean Difference (SMD) Between Add-on Regimen and Substitution Monotherapy Before and After Inverse Probability Treatment Weighting for all Covariates eFigure 5. Adjusted Time-to-Event Curves of Treatment Failure Based on Specific Antiseizure Medications (ASM) Used as Second Regimen eReferences [file jamanetwopen-e250354-s001.pdf]

Supplemental Online Content

Cerulli Irelli E, Cocchi E, Gesche J, et al. Second-line medications for women of childbearing age with idiopathic generalized epilepsy. *JAMA Netw Open*. 2025;8(3):e250354.
doi:10.1001/jamanetworkopen.2025.0354

eMethods
eTable 1. IPTW-Weighted Models for Treatment Failure Assuming a Linear Relationship Between Age and Substitution Monotherapy or Add-on
eTable 2. IPTW-Weighted Models for ASM Retention Assuming a Linear Relationship Between Age and Substitution Monotherapy/Add-on
eTable 3. IPTW-Weighted Models for Seizure Freedom Assuming a Linear Relationship Between age and Substitution Monotherapy or Add-on
eTable 4. Multivariable Cox Regression of Treatment Failure in Patients With Juvenile Absence Epilepsy
eTable 5. Multivariable Cox Regression of Treatment Failure in Patients With Juvenile Myoclonic Epilepsy
eTable 6. Multivariable Cox Regression of Treatment Failure in Patients With Epilepsy With Generalized Tonic-Clonic Seizures Alone
eTable 7. Multivariable Cox Regression of Treatment Failure Comparing Different ASMs
eTable 8. Multivariable Cox Regression of Treatment Failure Comparing Different ASM Combinations
eFigure 1. Missingness of Variables and Combinations of Missingness Using the Intersecting Sets Visualization Method
eFigure 2. Flowchart of Patient Selection, Second-Line Treatment Strategies, and Research Questions
eFigure 3. Prescribing Pattern of Substitution Monotherapy or Add-on Therapy Across Different Time Periods
eFigure 4. Love-Plot Showing Standardized Mean Difference (SMD) Between Add-on Regimen and Substitution Monotherapy Before and After Inverse Probability Treatment Weighting for all Covariates
eFigure 5. Adjusted Time-to-Event Curves of Treatment Failure Based on Specific Antiseizure Medications (ASM) Used as Second Regimen
eReferences

This supplemental material has been provided by the authors to give readers additional information about their work.

© 2025 Cerulli Irelli E et al. *JAMA Network Open*.

eMethods

Statistical analysis

Multiple imputation with chained equations was used to impute missing data, as explained in previous studies.[1] The pattern and rate of missing data are fully represented in eFigure1. Baseline characteristics between treatment groups were compared using Fisher's exact test for categorical variables, and unpaired t-test or Mann-Whitney U test for continuous variables, depending on their normal or non-normal distribution, respectively.

Consistent with our previous studies,[2,3] we applied inverse probability of treatment weighting (IPTW) to adjust for potential selection biases associated with second ASM assignment. IPTW helps to balance the distribution of observed covariates across different treatment groups, making the groups more comparable and thereby allowing for a more accurate estimation of treatment effect. The IPTW was calculated as the reciprocal of the probability of receiving either an add-on regimen or a substitution monotherapy, using a multivariable logistic regression model. Based on previous literature,[4-6] this model incorporated multiple covariates including age at second ASM prescription, febrile seizures, epilepsy syndrome (using GTCA as reference syndrome), catamenial worsening of seizures, history of status epilepticus, mild intellectual disability, and use of VPA as second ASM regimen. Age was treated non-linearly using a restricted cubic spline with knots at the 1st, 25th, 50th, 75th, and 99th percentiles, based on findings from previous studies on the same cohort and to mitigate potential effects of linearity assumption violations.[2,3,7] The standardized mean differences (SMD) pre- and post-IPTW adjustment are reported, illustrating the balance of baseline characteristics between the treatment groups.

An IPTW-adjusted Cox proportional hazards model was employed to assess the differences in time to TF between substitution monotherapy and add-on as second ASM regimen. The time of entry was the date of second ASM prescription, and the time of endpoint was the date of TF or the last follow-up visit, truncated at 5 years of follow-up. To address potential differences related to the year of prescription over the extended study timespan, a sensitivity analysis was conducted using a mixed

© 2025 Cerulli Irelli E et al. *JAMA Network Open.*

IPTW-adjusted Cox proportional hazards model, with the year of prescription incorporated as a random effect. For this analysis, the year was categorized into five-year intervals to reduce the number of levels, with the 1995–1999 and 2000–2004 periods combined into a single interval due to the limited number of patients in these periods. Additionally, a further sensitivity analysis of the primary outcome was performed, after excluding patients using VPA as second ASM.

Regarding secondary outcome measures, the same IPTW-weighted Cox proportional hazards model was used to analyze the differences in ASM retention. For estimating differences in seizure freedom between the two ASM regimens, an IPTW-weighted binary logistic regression analysis was conducted, with follow-up duration included as an additional covariate to account for variations in follow-up.

Additionally, we compared: 1) TF due to ineffectiveness based on specific ASMs prescribed after the failure of the first-line monotherapy, assessed through multivariable Cox regression, using the type of ASM regimen (i.e., substitution monotherapy or add-on) and epilepsy syndrome as covariate. To ensure more reliable estimates, only ASMs prescribed to at least 10 patients were included in this analysis; 2) TF based on specific ASM combinations among patients using add-on regimens, assessed through multivariable Cox regression analysis and including epilepsy syndrome as covariate; 3) TF according to specific IGE syndromes, using multivariable Cox regression, adjusted for the prescribed ASM. Due to the relatively small patient subgroups in these exploratory analyses, a complete case analysis was performed to avoid introducing potential bias from multiple imputations.

Finally, the proportion of patients experiencing ASM discontinuation due to adverse effects in the two treatment groups was compared through Fisher's exact test. Two-sided P values less than 0.05 were considered statistically significant.

All analyses in this study were conducted using R version 4.3.3.

© 2025 Cerulli Irelli E et al. *JAMA Network Open*.

**eTable 1. IPTW-Weighted Models for Treatment Failure Assuming a Linear Relationship Between Age and Substitution Monotherapy or Add-on**			
	Hazard ratio	95% CI	p value
Add-on therapy vs substitution monotherapy	0.89	0.54-1.47	0.65
Abbreviations: CI = confidence interval of beta coefficients. IPTW = Inverse probability of treatment weighting			

**eTable 2. IPTW-Weighted Models for ASM Retention Assuming a Linear Relationship Between Age and Substitution Monotherapy/Add-on**			
	Hazard ratio	95% CI	p value
Add-on therapy vs substitution monotherapy	0.94	0.56-1.58	0.82
Abbreviations: CI = confidence interval of beta coefficients. IPTW = Inverse probability of treatment weighting			

**Etable 3. IPTW-Weighted Models for Seizure Freedom Assuming a Linear Relationship Between age and Substitution Monotherapy or Add-on**			
	Odds ratio	95% CI	p value
Add-on therapy vs substitution monotherapy	0.79	0.46-1.36	0.40
Abbreviations: CI = confidence interval of beta coefficients. IPTW = Inverse probability of treatment weighting			

**Etable 4. Multivariable Cox Regression of Treatment Failure in Patients With Juvenile Absence Epilepsy**			
	Hazard ratio	95% CI	p value
Add-on therapy vs substitution monotherapy	1.53	0.56-4.18	0.40
Valproate as second ASM (reference)			
Levetiracetam used as second ASM	1.37	0.29-6.44	0.69
Lamotrigine used as second ASM	2.29	0.49-10.67	0.29
Topiramate used as second ASM	3.88	0.76-19.71	0.10
Sodium channel blocker used as second ASM	3.95	0.38-41.28	0.25
Other medication used as second ASM	6.29	1.63-24.20	0.008
Abbreviations: ASM= antiseizure medication; CI = confidence interval.			

© 2025 Cerulli Irelli E et al. *JAMA Network Open.*

**eTable 5. Multivariable Cox Regression of Treatment Failure in Patients With Juvenile Myoclonic Epilepsy**			
	Hazard ratio	95% CI	p value
Add-on therapy vs substitution monotherapy	0.81	0.41-1.63	0.56
Valproate as second ASM (reference)			
Levetiracetam used as second ASM	3.72	0.53-25.75	0.18
Lamotrigine used as second ASM	7.93	1.15-54.47	0.035
Topiramate used as second ASM	7.81	0.94-64.69	0.06
Sodium channel blocker used as second ASM	28.09	3.42-230.63	0.002
Other medication used as second ASM	7.35	0.84-63.96	0.07
Abbreviations: ASM= antiseizure medication; CI = confidence interval.			

**eTable 6. Multivariable Cox Regression of Treatment Failure in Patients With Epilepsy With Generalized Tonic-Clonic Seizures Alone**			
	Hazard ratio	95% CI	p value
Add-on therapy vs substitution monotherapy	0.27	0.06-1.19	0.08
Valproate as second ASM (reference)			
Levetiracetam used as second ASM	0.22	0.02-2.21	0.20
Lamotrigine used as second ASM	0.55	0.01-3.06	0.49
Topiramate used as second ASM	0.36	0.037-3.48	0.37
Sodium channel blocker used as second ASM	0.95	0.14-6.46	0.95
Other medication used as second ASM	3.27	0.66-16.24	0.15
Abbreviations: ASM= antiseizure medication; CI = confidence interval.			

© 2025 Cerulli Irelli E et al. *JAMA Network Open*.

eTable 7. Multivariable Cox Regression of Treatment Failure Comparing Different ASMs			
	Hazard ratio	95% CI	p value
Valproate as second ASM (reference)			
Levetiracetam used as second ASM	1.69	0.66-4.35	0.27
Lamotrigine used as second ASM	2.99	1.18-7.57	0.02
Topiramate used as second ASM	3.21	1.10-9.38	0.03
Sodium channel blocker used as second ASM	9.13	2.97-28.1	<0.001
Add-on therapy vs substitution monotherapy	0.74	0.44-1.26	0.27
Epilepsy syndrome			
Absence epilepsy (reference)			
Juvenile myoclonic epilepsy	1.53	0.82-2.86	0.18
Epilepsy with generalized tonic-clonic seizures alone	0.62	0.27-1.42	0.26
Abbreviations: ASM= antiseizure medication; CI = confidence interval.			

eTable 8. Multivariable Cox Regression of Treatment Failure Comparing Different ASM Combinations			
	Hazard ratio	95% CI	p value
Levetiracetam + lamotrigine (reference)			
Valproate + lamotrigine or levetiracetam	0.14	0.02-1.07	0.057
Levetiracetam + other ASM	2.41	1.12-5.17	0.02
Lamotrigine + other ASM	4.03	1.73-9.39	0.001
Epilepsy syndrome			
Absence epilepsy (reference)			
Juvenile myoclonic epilepsy	0.98	0.48-2.01	0.96
Epilepsy with generalized tonic-clonic seizures alone	0.38	0.16-0.93	0.038
Abbreviations: ASM = antiseizure medication; CI = confidence interval.			

© 2025 Cerulli Irelli E et al. *JAMA Network Open*.

eFigure 1. Missingness of Variables and Combinations of Missingness Using the Intersecting Sets Visualization Method

Figure legend: Only variables with at least one missing observation are represented in the figure. The number of observations missing for each variable is expressed as Set size. Intersection plots in the lower part of the figure link different variables missing together in the same participant, whereas the number of times the data is missing alone or in combination is expressed as intersection size.

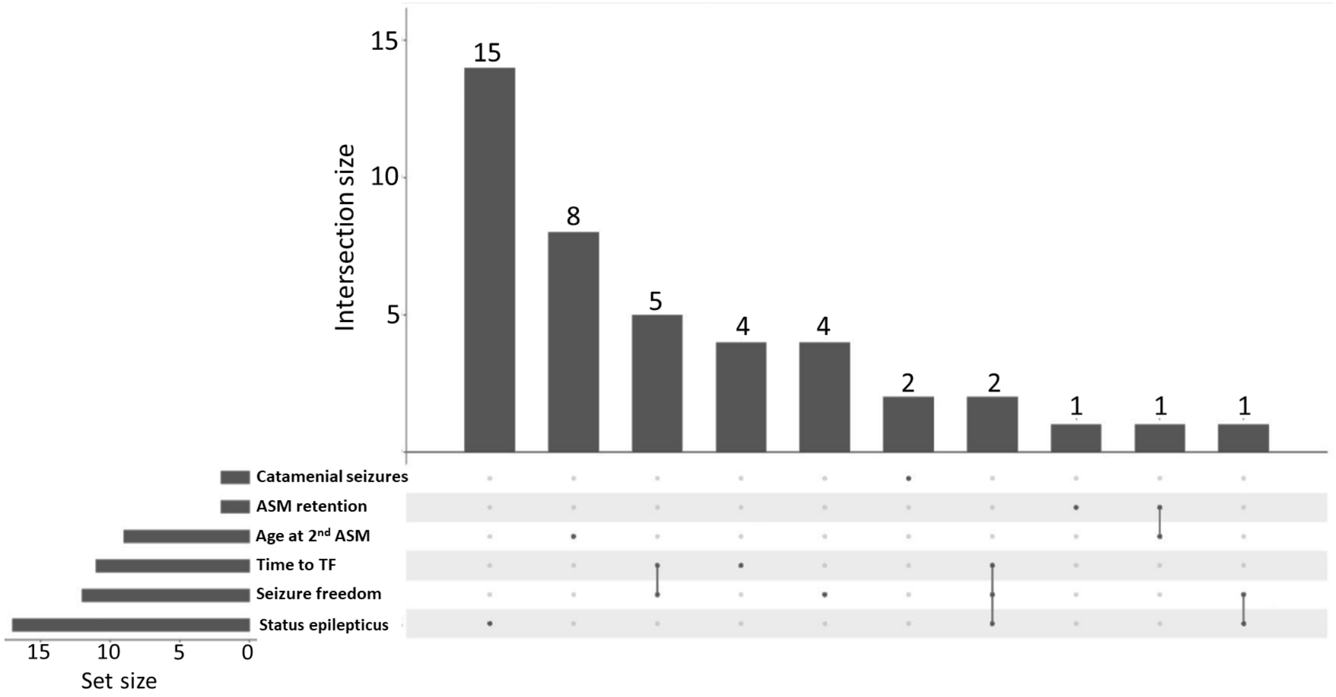

© 2025 Cerulli Irelli E et al. *JAMA Network Open*.

eFigure 2. Flowchart of Patient Selection, Second-Line Treatment Strategies, and Research Questions

Abbrevations: ASM = antiseizure medication; IGE = idiopathic generalized epilepsy; LEV = levetiracetam; LTG = lamotrigine; TPM = topiramate; VPA = valproate.

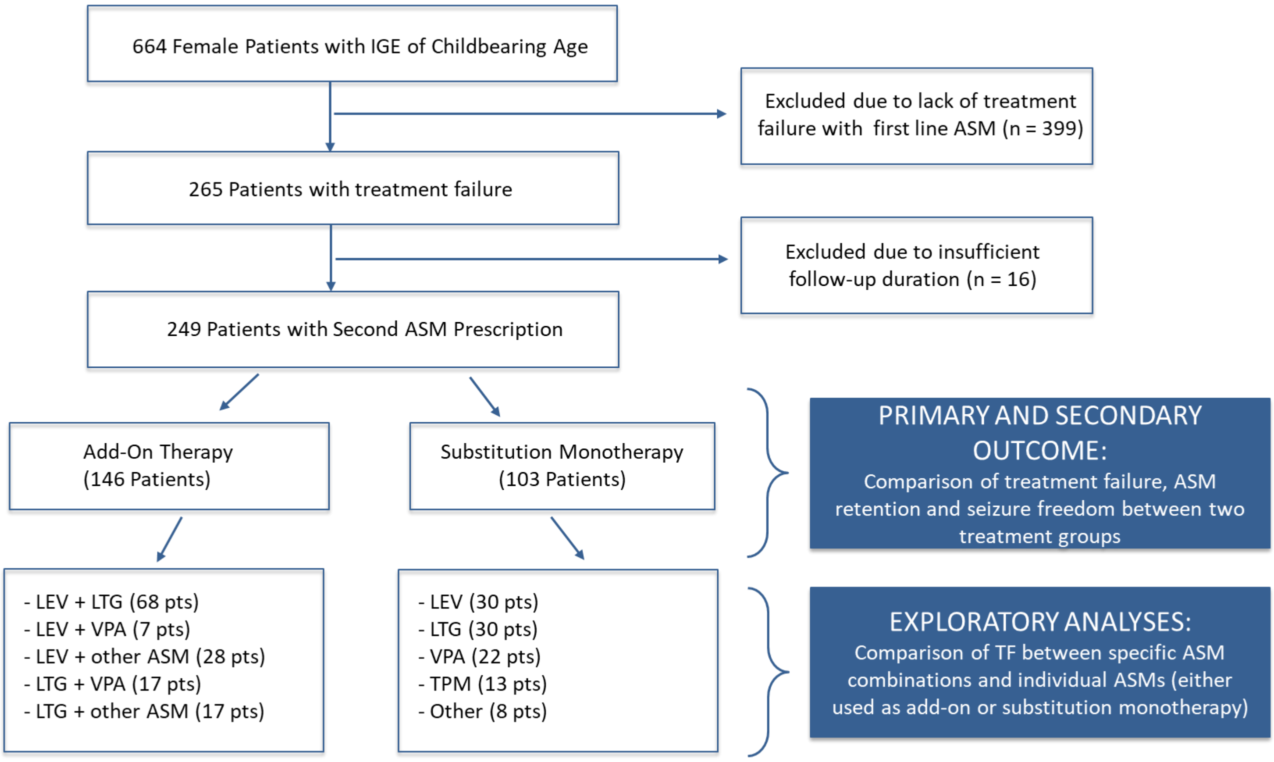

© 2025 Cerulli Irelli E et al. *JAMA Network Open*.

eFigure 3. Prescribing Pattern of Substitution Monotherapy or Add-on Therapy Across Different Time Periods

The percentage of patients receiving either substitution monotherapy or add-on therapy is represented by colored bars, indicating the proportion of patients in each category. Time periods are shown along the x-axis, with the number of patients in each period provided in brackets. Due to the low number of patients in the 1995-1999 and 2000-2004 periods (5 and 16 patients, respectively), these time periods have been combined into a single period for this figure.

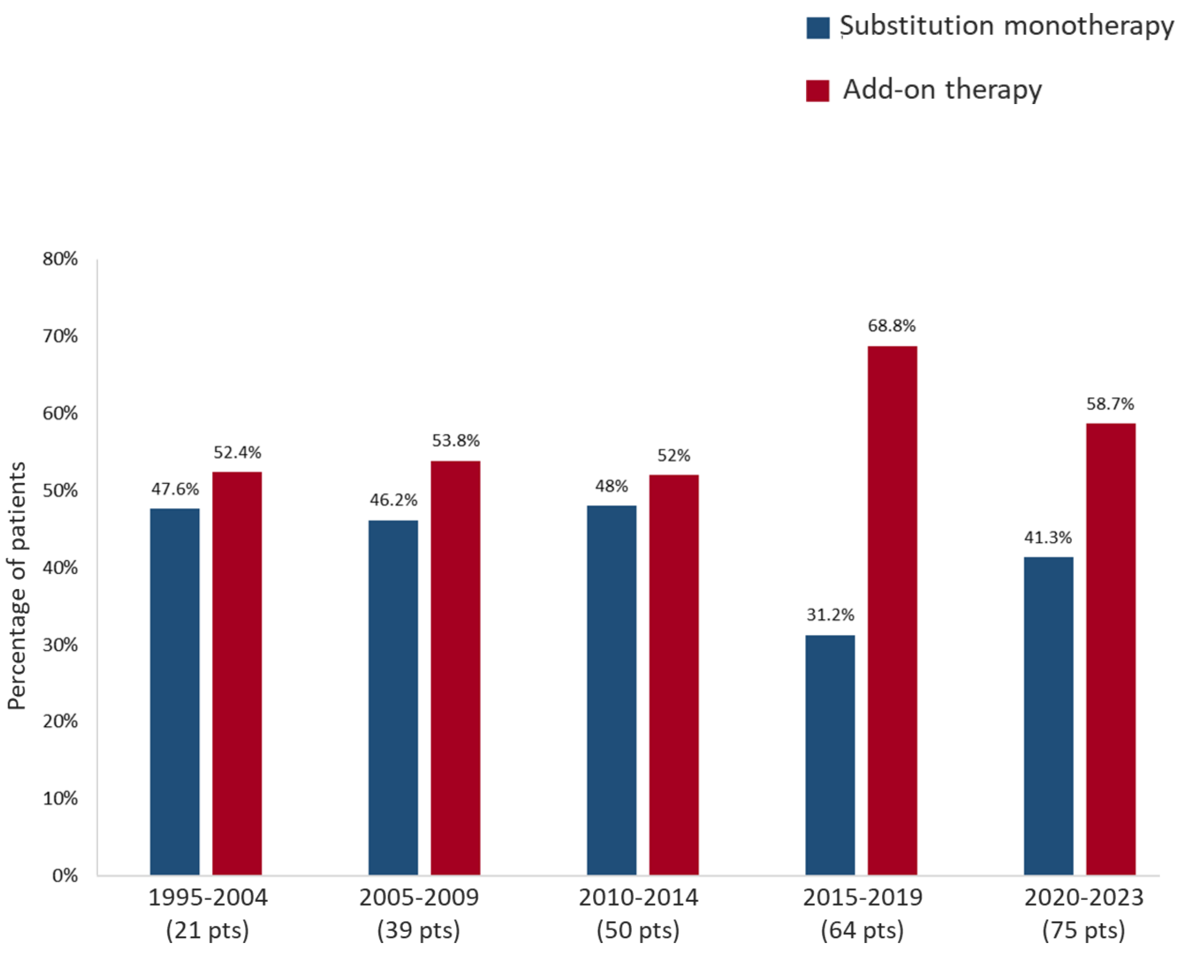

© 2025 Cerulli Irelli E et al. *JAMA Network Open*.

eFigure 4. Love-Plot Showing Standardized Mean Difference (SMD) Between Add-on Regimen and Substitution Monotherapy Before and After Inverse Probability Treatment Weighting for all Covariates

Figure legend: the vertical dotted line represents the interval of 0.1 of SMD within which balance is considered acceptable

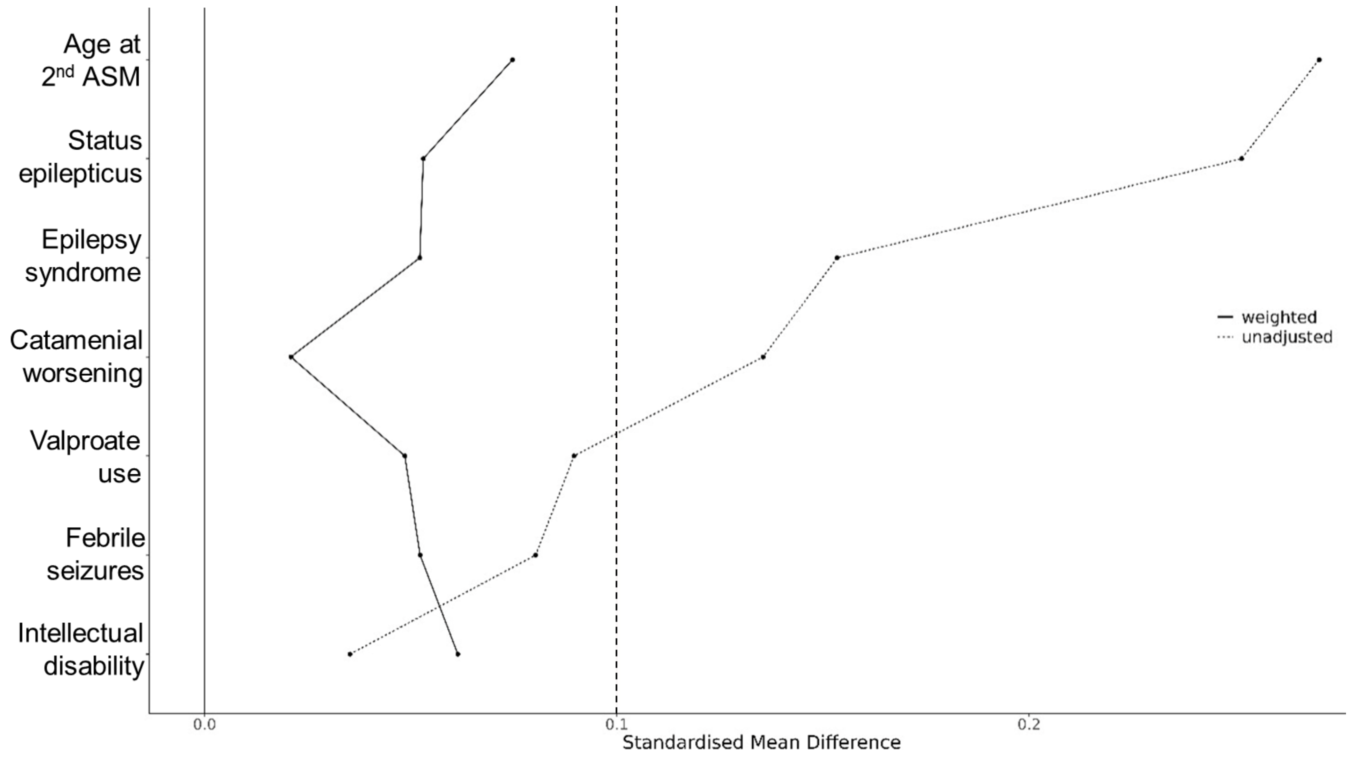

© 2025 Cerulli Irelli E et al. *JAMA Network Open*.

eFigure 5. Adjusted Time-to-Event Curves of Treatment Failure Based on Specific Antiseizure Medications (ASM) Used as Second Regimen

This graph represents survival curves for treatment failure based on specific ASM used as the second regimen, either as an add-on or substitution monotherapy, adjusted for epilepsy syndrome and the type of regimen used. Each ASM is represented by a specific color, along with hazard ratios and 95% confidence intervals compared with reference ASM (i.e., valproate). Abbreviations: AZM = acetazolamide; ESM = ethosuximide; LEV = levetiracetam; LTG = lamotrigine; PER = perampanel; PB = phenobarbital; SCB = sodium channel blocker (8 lacosamide, 3 carbamazepine); TPM = topiramate; VPA = valproate; ZNS = zonisamide.

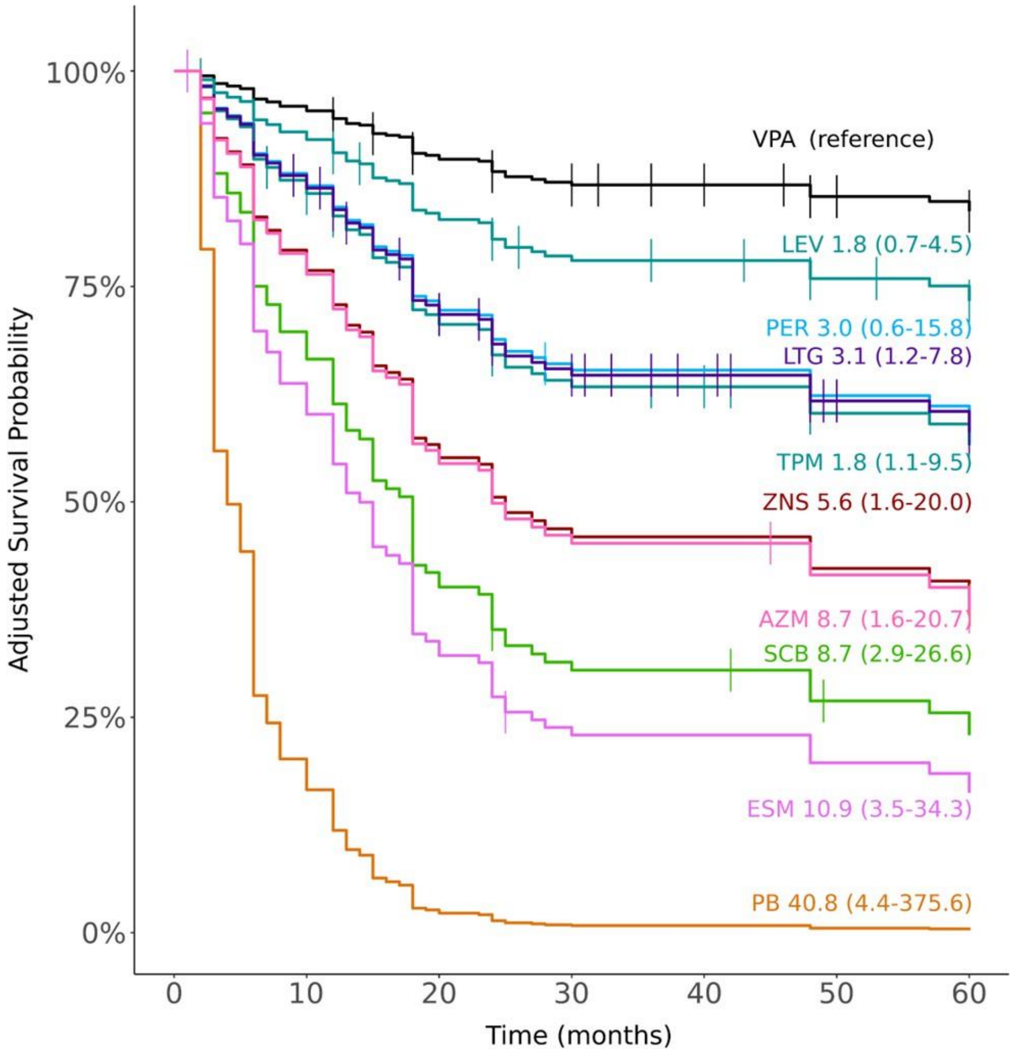

eReferences

1. Moons KG, Donders RA, Stijnen T, Harrell FE Jr. Using the outcome for imputation of missing predictor values was preferred. J Clin Epidemiol. 2006;59(10):1092-1101. doi:10.1016/j.jclinepi.2006.01.009

2. Cerulli Irelli E, Cocchi E, Morano A, et al. Levetiracetam vs Lamotrigine as First-Line Antiseizure Medication in Female Patients With Idiopathic Generalized Epilepsy. *JAMA Neurol*. 2023;80(11):1174-1181. doi:10.1001/jamaneurol.2023.3400

3. Cerulli Irelli E, Cocchi E, Gesche J, et al. Lamotrigine vs levetiracetam in female patients of childbearing age with juvenile absence epilepsy: A Bayesian reanalysis. *Epilepsia*. Published online August 10, 2024. doi:10.1111/epi.18087

4. Cerulli Irelli E, Gesche J, Schlabitz S, et al. Epilepsy with generalized tonic-clonic seizures alone: Electroclinical features and prognostic patterns. *Epilepsia*. 2024;65(1):84-94. doi:10.1111/epi.17809

5. Cerulli Irelli E, Morano A, Orlando B, et al. Seizure outcome trajectories in a well-defined cohort of newly diagnosed juvenile myoclonic epilepsy patients. *Acta Neurol Scand*. 2022;145(3):314-321. doi:10.1111/ane.13556

6. Ayoub D, Jaafar F, Al-Hajje A, et al. Predictors of drug-resistant epilepsy in childhood epilepsy syndromes: A subgroup analysis from a prospective cohort study. *Epilepsia*. Published online August 16, 2024. doi:10.1111/epi.18100

7. Cerulli Irelli E, Cocchi E, Morano A, et al. Predictors of Seizure Recurrence in Women With Idiopathic Generalized Epilepsy Who Switch From Valproate to Another Medication. *Neurology*. 2024;102(9):e209222. doi:10.1212/WNL.0000000000209222

© 2025 Cerulli Irelli E et al. *JAMA Network Open*.
