# Supplementary material for: Second-Line Medications for Women Aged 10 to 50 Years With Idiopathic Generalized Epilepsy
Source: JAMA Netw Open. 2025 Mar 10;8(3):e250354. doi: 10.1001/jamanetworkopen.2025.0354 (PMC11894492; doi:10.1001/jamanetworkopen.2025.0354)
Supplement: Supplement 2. — Nonauthor Collaborators [file jamanetwopen-e250354-s002.pdf]

Supplemental Online Content: Nonauthor Collaborators
*First name, last name, and suffix (if applicable) are required and will appear in PubMed.

***Group Name(s): Women with Epilepsy Treatment Options and Research (WETOR) study group**							
***First Name and Middle Initial(s)**	***Last Name**	***Suffix (eg, Jr, III)**	Academic Degrees	Institution	Location (city, state/province, country)	Role or Contribution, eg, chair, principal investigator	Group (if more than 1 Group listed in the byline) and/or Subgroup (eg, Steering Committee)
Betul	Baykan			Istanbul University Istanbul Faculty of Medicine, Department of Neurology and EMAR Medical Center, Istanbul Turkey		Site investigator	
Daniela	Fatuzzo			AOU Policlinico G. Rodolico – San Marco, Catania, Italy		Site investigator	
Giulio	Pastorelli			Neuromuscular and Sense Organs Department, Careggi University Hospital, Florence, Italy.		Site investigator	
Pierpaolo	Quarato			IRCCS NEUROMED, Pozzilli, Isernia, Italy.		Site investigator	
Cecilia	Catania			EEG and Epilepsy Unit, Department of Neuroscience, University Hospital and Faculty of Medicine of Geneva, University of Geneva, Geneva, Switzerland		Site investigator	
Sara	Casciato			Department of Neurosciences, S. Camillo-Forlanini Hospital, Rome, Italy		Site investigator	
Chiara	Martellino			Neurophysiopatology and Movement Disorders Clinic, University of Messina, Italy		Site investigator	
Giorgia	Atanasio			Neurophysiopatology and Movement Disorders Clinic, University of Messina, Italy		Site investigator	
Emanuela	Viglietta			Humanitas Gradenigo Hospital, Turin, Italy.		Site investigator	
Vittoria	Cianci			Great Metropolitan Hospital "Bianchi-Melacrino-Morelli", Reggio Calabria, Italy.		Site investigator	
Irene	Bagnasco			Martini Hospital, Turin, Italy		Site investigator	

Supplemental Online Content: Nonauthor Collaborators
*First name, last name, and suffix (if applicable) are required and will appear in PubMed.

***First Name and Middle Initial(s)**	***Last Name**	***Suffix (eg, Jr, III)**	Academic Degrees	Institution	Location (city, state/province, country)	Role or Contribution, eg, chair, principal investigator	Group (if more than 1 Group listed in the byline) and/or Subgroup (eg, Steering Committee)
Erica	Cognolato			Martini Hospital, Turin, Italy		Site investigator	
Edoardo	Pronello			Neurology Unit, Department of Translational Medicine, University of Piemonte Orientale, Novara		Site investigator	
Francesca	Bisulli			IRCCS Istituto delle Scienze Neurologiche di Bologna, Bologna, Italy; DIBINEM, University of Bologna, Bologna, Italy		Site investigator	
Giuseppe	Pontrelli			"SS. Annunziata Hospital", Taranto, Italy		Site investigator	
Giovanni	Boero			"SS. Annunziata Hospital", Taranto, Italy		Site investigator	
Francesca	Gilio			Sandro Pertini Hospital, Rome, Italy		Site investigator	
Francesca	Gragnani			Sandro Pertini Hospital, Rome, Italy		Site investigator	
Carla	Marini			Child Neurology and Psychiatric Unit, Pediatric Hospital G. Salesi, United Hospitals of Ancona, Ancona, Italy.		Site investigator	
Sara	Gasparini			Department of Medical and Surgical Sciences, Magna Graecia University of Catanzaro, Catanzaro, Italy; Great Metropolitan Hospital "Bianchi-Melacrino-Morelli", Reggio Calabria, Italy.		Site investigator	
Irene	Pappalardo			Clinical Neurophysiology and Epilepsy Unit, IRCCS San Martino Hospital, Genova, Italy.		Site investigator	
