# Supplementary material for: Second-Line Medications for Women Aged 10 to 50 Years With Idiopathic Generalized Epilepsy
Source: JAMA Netw Open. 2025 Mar 10;8(3):e250354. doi: 10.1001/jamanetworkopen.2025.0354 (PMC11894492; doi:10.1001/jamanetworkopen.2025.0354)
Supplement: Supplement 3. — Data Sharing Statement [file jamanetwopen-e250354-s003.pdf]

Data Sharing Statement

Cerulli Irelli. Second-Line Medications for Women of Childbearing Age With Idiopathic Generalized Epilepsy. *JAMA Netw Open*. Published March 10, 2025.
doi:10.1001/jamanetworkopen.2025.0354

Data
Data available: No

Additional Information
Explanation for why data not available: Completely anonymized data will be available to qualified academic investigators to replicate study results by reasonable request. Data transfer will be regulated by material transfer agreements.
